# Characterizing the Collective Personality of Ant Societies: Aggressive Colonies Do Not Abandon Their Home

**DOI:** 10.1371/journal.pone.0033314

**Published:** 2012-03-21

**Authors:** Inon Scharf, Andreas P. Modlmeier, Stephan Fries, Claire Tirard, Susanne Foitzik

**Affiliations:** 1 Institute of Zoology, Johannes Gutenberg University of Mainz, Mainz, Germany; 2 Laboratoire Ecologie & Evolution CNRS UMR 7625, Université Pierre et Marie Curie, Paris 6, Paris, France; Cajal Institute, Consejo Superior de Investigaciones Científicas, Spain

## Abstract

Animal groups can show consistent behaviors or personalities just like solitary animals. We studied the collective behavior of *Temnothorax nylanderi* ant colonies, including consistency in behavior and correlations between different behavioral traits. We focused on four collective behaviors (aggression against intruders, nest relocation, removal of infected corpses and nest reconstruction) and also tested for links to the immune defense level of a colony and a fitness component (per-capita productivity). Behaviors leading to an increased exposure of ants to micro-parasites were expected to be positively associated with immune defense measures and indeed colonies that often relocated to other nest sites showed increased immune defense levels. Besides, colonies that responded with low aggression to intruders or failed to remove infected corpses, showed a higher likelihood to move to a new nest site. This resembles the trade-off between aggression and relocation often observed in solitary animals. Finally, one of the behaviors, nest reconstruction, was positively linked to per-capita productivity, whereas other colony-level behaviors, such as aggression against intruders, showed no association, albeit all behaviors were expected to be important for fitness under field conditions. In summary, our study shows that ant societies exhibit complex personalities that can be associated to the physiology and fitness of the colony. Some of these behaviors are linked in suites of correlated behaviors, similar to personalities of solitary animals.

## Introduction

Variation in heritable traits such as morphology or behavior is expected to be constantly removed from natural populations by drift and natural or sexual selection and it is therefore interesting to study the factors that maintain variation [1], [2]. Behavioral syndromes, defined as the consistency in behavior across different situations and contexts, can explain why behavioral variation is kept. The same behavior may be beneficial in certain contexts but may be maladaptive in other situations (e.g., [3]). This could result in non-directional selection on distinct behavioral types. For example, aggression can be useful against prey or competitors, but it can discourage or even lead to the premature death of potential mates, as it has been shown in a fishing spider [4]. In addition, being active in the absence of predators may be beneficial, but high activity levels in their presence are often risky [5]. Therefore, detecting correlations between behavioral traits is valuable for understanding how intra-population variation is maintained.

Behavioral syndromes have been described in various animal systems, with recurrent correlations between certain behaviors. A common behavioral syndrome is the aggressiveness-boldness syndrome. Aggressive individuals often tend to be more active and they take more risks (e.g., [5], [6]). For instance, aggressive male field crickets went faster out of refuge in a novel environment, that is, they were also bolder [7]. Another common behavioral syndrome is related to activity in general. Many behaviors tend to be positively associated with the activity level of an individual, because they are simultaneously affected by the metabolic rate or time constraints [6], [8]. This should lead to a triplet positive association among aggressiveness, boldness and general activity. Behavioral syndromes in solitary animals are often linked to physiological or life-history traits of the organism, which can help understanding the proximate correlates of behavior. For example, metabolic rate differences are a possible physiological explanation for consistent inter-individual differences in activity and aggression, explaining in part the aggressiveness-boldness-activity syndrome [6], [8].

While behavioral correlations are well documented for some solitary animals, there is still little evidence for behavioral correlations in animal societies (but see three recent papers: [9], [10], [11]). Using behavioral methodologies typical for solitary animals, these studies deal with research questions relevant to social insects, such as behavioral differences between castes or cooperative behavior of the whole colony. For example, Chapman et al. [9] showed that the patroller caste in *Myrmica* ants exhibited the common aggressiveness-boldness syndrome, while the brood-carer caste did not. In addition, the behavior of these two castes is correlated on the colony level (i.e., the whole colony is sometimes more aggressive and bold). Interestingly, social groups, as a whole, may differ in behavior, which can affect their success. Wray et al. [11] showed that the defensive response of honey bee colonies was correlated with fitness components (the colony weight). In social insects, selection predominantly acts on the colony level and collective behaviors such as communal defense, networking in foraging and nest construction are expected to be strongly linked to colony productivity. Hence, colony behavior can be shaped by natural selection similar to the behavior of multicellular organisms.

We used colonies of the European cavity-dwelling ant, *Temnothorax nylanderi*, to study consistency, variation and the relationship of four important behaviors: (1) aggression towards an intruder, (2) nest relocation, (3) removal of an infected corpse, and (4) nest reconstruction after partial destruction. These behaviors represent important activities of animal societies in general. Collective defense is typical for many groups, and is evident in many bird species living in groups, as mobbing of predators gets more efficient with colony size [12]. In social insects, aggression is vital in defending the nest and has fitness consequences (e.g., [11]). Collective movement is an important trait of animal groups expressed by fish schools, bird flocks, locust swarms and social insects (reviewed in [13]). Nest relocation is a common behavior in many social insect species, which is exhibited when the present nest becomes unsuitable for some reason, such as decomposition of the nesting material in cavity-dwelling ants [14], [15] or local food depletion in army ants (e.g., [16]). Other reasons for nest relocation are reproduction – a large colony splits into two parts and one of them leaves, i.e., reproduction by budding [15] – and simply moving into a better-larger nest [17].

Living in groups increases the parasite burden and the risk of infection by contact-transmitted parasites [12], [18]. Therefore, removal of waste and corpses of dead group members is crucial for colony health [19]. Indeed, waste management has been found in various group-living animals, which live in the same nest sites for longer time periods (e.g., aphids, mites and ants [20], [21], [22]). Nest sites provide a safe environment to raise offspring, but they have to be constructed and maintained, and failure to repair may lead to exposure to external risks, such as predators or parasites [23]. In order to increase defensiveness against intruders, colonies often block or reduce the nest entrance by using soil, sand or wooden pieces (e.g., [14], [24], [25]). Moreover, colonies of the ant genus *Temnothorax* prefer nest sites with very small entrances so that a single ant could control colony entry [14].

Even before testing behavior under different conditions, testing for repeatability of behavior under the same conditions is a necessary step in characterizing behavioral syndromes and personality [26]. Second, searching for collective colony personality, we looked for positive and/or negative correlations among the four behavioral traits. We predict that similar to the aggressiveness-boldness-activity syndrome in solitary animals, aggressive colonies should be bolder and more active. Therefore, they should show a better performance in the other collective behaviors such as nest reconstruction. However, as ant colonies are also energy limited we expect some behavioral trade-offs. In addition, it is intriguing to relate behavior and personality to fitness components [26], [27], and to understand whether different colony personalities result in the same final fitness. We therefore tested how the four behaviors correlate with per-capita productivity as a measure of colony efficiency (e.g., [28], [29]). The four documented behaviors are important for colony survival, representing ways to overcome stress and threat. We expect in general a positive effect of the measured behaviors on per-capita productivity. However, we do not expect a perfect match, because the same goal can be achieved in parallel ways, and specializing in one behavior may lead to another becoming superfluous.

Immune defense is an important physiological trait in social insects, because frequent interactions of genetically similar individuals lead to a great risk of contagious infections [18], [19]. The level of immune defense may correlate with inter-colony differences in some behaviors, such as corpse removal and nest relocation. Encounters with infected corpses pose a direct threat to the colony, and the ants should react by increasing their immune defense [22]. However, ant colonies that recognize and remove infectious material from the nest faster and thus show a high social immunity, might be able to invest less in the physiological immune defense. Nest relocation may be triggered by exposure to parasites, but during the move ants and their brood are also vulnerable to infection and predation [19]. Similarly, intruders might increase micro-parasite exposure [18]. We therefore expected that colonies which show a high tendency to expose their members to parasites either during nest migration, nest defense or by failing to reconstruct their nest site should invest highly in their immune function. In contrast, ant colonies that remove infected corpses fast from the nest are expected to show low immune functions. Nest relocation should show the best positive correlation with the immune defense level, because during emigrations all colony members are exposed to the surrounding environment.

## Methods

### Study organisms and sites

*Temnothorax nylanderi* are small cavity-dwelling ants inhabiting forests in western and central Europe. Their colonies comprise several dozen workers and a single queen. They reside in preformed cavities of various wooden structures on the forest floor providing protection from the outer environment [30], [31]. Due to the decomposition of their nests in the field, these ant colonies are forced to frequently relocate their nests [14], [17]. *Temnothorax nylanderi* is a suitable species for the research questions because it shows high variability in behavior, mainly in relocation and aggressive tendencies. As mentioned, all studied behaviors have implications for the colony's performance under natural conditions. We collected 50 *T. nylanderi* colonies in summer 2009 in Sommerhausen, Germany (49.706N, 10.030E). No specific permits were required for the described ant collection (the collection site is not privately owned or protected and the collected ants are not endangered or protected). The colonies were brought to the laboratory and moved to artificial nests (7.5×2.5×0.5 cm) in plastic boxes (10×10×1.5 cm) with a plastered floor. The nests were kept in climate chambers imitating the natural temperatures around the year in the habitat of origin (summer: 20°/15°C day/night; autumn/spring: 15°/10°C day/night; winter: temperatures gradually decreased to −5°C). Colonies were fed weekly with honey, crickets and water. Colonies were kept for ∼1.5 years under standard conditions to moderate environmental effects on behavior, physiology and life-history traits. Consequently, we estimate that more than 50% of the workers emerged in the laboratory. Two weeks prior to the experiment, colonies were moved to summer conditions. The experiments were conducted at room temperature.

### Experimental design

In March–May 2011 we tested the performance of the ant colonies in four standardized tests. We were interested in four important behaviors: (I) aggression against a conspecific intruder, (II) nest relocation, (III) corpse removal out of the nest, and (IV) reconstruction of the nest after partial opening. Each behavior was tested twice for each colony, with one week in-between experiments. The behavioral observations were performed under stereomicroscopes by the two first authors, but all behaviors of a colony were observed by the same observer. In addition, we counted the number of workers (i.e., colony size) before the first behavioral test. After the aggression tests, we counted the number of brood items: New queen, male and worker pupae, pre-pupae and larvae and calculated the per-capita productivity for each colony (colony production divided by colony size). We were also interested in possible correlations of those four behaviors with the immune defense level.

#### I. Aggression against a conspecific intruder

Colony aggression was quantified by entering a dead non-nestmate conspecific worker into the nest and measuring all aggressive interactions during the next five minutes (according to the protocol described in [32], [33]). Specifically, we documented every 20–30 seconds how many ants were either antennating or attacking the intruder (11 observations per trial). Dead ants, killed by freezing in −20°C prior to the experiment, were used to eliminate behavioral variation between the stimuli and focus on the focal colony's response to the chemical stimulus. We used the percent of aggressive interactions (no. of mandible spreading, biting, dragging, holding and stinging events divided by all of the above plus antennating events), as a measure of aggression [32], [33]. Aggression tests were performed twice for each colony (one week between the two trials).

#### II. Nest relocation

On the day after each aggression test, we gave the ants the opportunity to relocate into new nests, without damaging the original nest. The new nest, which was identical to the original one, was placed seven centimetres from the occupied nest, and we noted after 24 hours whether the colonies relocated. It was a reasonable time, since after 24 hours ∼40% of the colonies moved at least three workers to the new nest. We scored the relocation tendency according to four levels: No relocation (0), three or more workers without brood occur in the new nest (1), workers and brood occur in the new nest but still also occupy the old one, i.e., the colony has split (possible in *T. nylanderi*) (2), and complete relocation, the old nest is empty (3).

#### III. Removal of a corpse out of the nest

**Figure 1:**
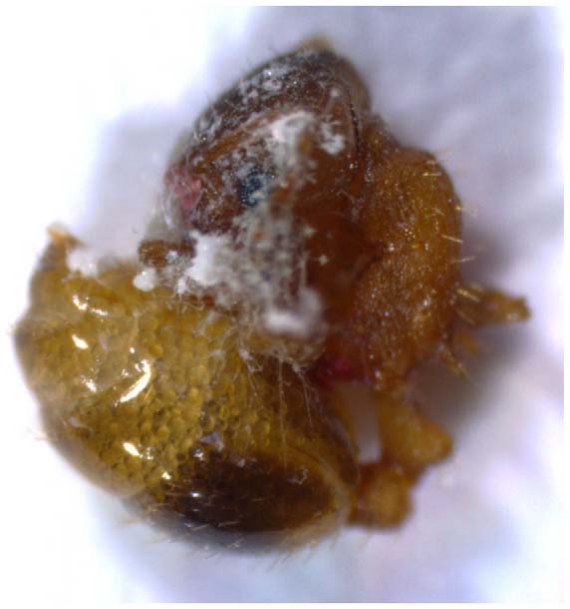
A decaying corpse of a *T. nylanderi* ant covered by unidentified fungi, which was used for the corpse removal experiment. A conspecific in such a condition presumably poses some risk of micro-parasite infection to the colony and should be removed from the nest.

Three weeks later we sacrificed a large *T. nylanderi* colony by freezing at −20°C, opened its nest, added some water and let the ants decompose for 48 hours at room temperature (20°C). After that time the ant corpses were covered by white unidentified fungi (Fig. 1). We entered three ant corpses (randomly chosen) to each nest and measured the time the colony required to remove the first dead decomposed ant. We obtained the exact time of the corpse removal, if it was removed within the first 16 minutes. We took another observation after 30 minutes. If the corpse was removed after 16 minutes and before 30 minutes, the colony received the value “23 minutes”, and if no corpse was removed after 30 minutes, the colony received the maximal value of “30 minutes” (occurred in 5% of the cases). We repeated this procedure a week later.

#### IV. Nest reconstruction after partial opening

**Figure 2:**
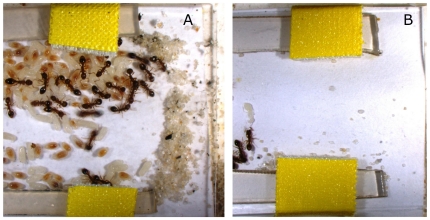
Two *T. nylanderi* nests representing two extremes of nest reconstruction after partial opening: (A) entrance is almost fully blocked again; (B) entrance is almost unblocked. Entrance blocking should protect better against invasions to the nest.

Two weeks after the corpse removal experiment we measured the last behavior, the reconstruction of the nest. We first removed parts of the plastic nest, so that the nest entrance was increased from 0.3 cm to 1.5 cm. We provided the ant nests with a standardized amount of fine sand (1 ml), which could be used by the workers to block part of the now widened nest entrance. Previous experiments with *Temnothorax* ants have shown that these ants prefer nest sites with small entrances, which are easier to defend against larger ants [14]. Two days later we photographed the nest entrance and measured the percentage of the entrance which was covered with sand particles (Fig. 2): We drew a line closing the entrance and calculated the proportion of this line covered with sand. Digital measurements were done by the first author using the software ImageJ. We repeated the whole procedure a week later.

#### V. Immune defense level

After the behavioral experiments, we randomly chose 21 colonies and collected two foragers, two ants located next to the entrance and two ants taking care of the brood from each colony for immune defense measurements (a total of six ants, representing the colony immune defense level). We chose ants performing different tasks, as a previous study pointed to differences in immune defense among castes or workers performing different tasks (e.g., [34]). As a proxy of immune defense level, we measured the activity of the phenoloxidase enzyme (PO). This enzyme is mainly found in the hemolymph as pro-phenoloxidase (PPO) and is activated into phenoloxidase prior to measurement. We therefore measured the total amount of PO potentially available for the individual (both activated and stored pro-enzyme). PO is an important component of invertebrates' immune defense and is involved in melanisation, cellular defense response and wound healing processes [35].

We used a similar procedure to Bocher et al. [34]. We placed individual ants in 20 µL sodium cacodilate/CaCl_2_ buffer (0.01 M Na-Cac, 0.005 M CaCl_2_), cut them with small scissors, and centrifuged using a cooling device. We moved the liquid part to new Eppendorf tubes and added 10 µL chymotrypsin to activate the PPO. As a substrate we used L-DOPA (4 mg mL^−1^ in distilled water) and the reaction was performed at 30°C in a temperature-controlled spectrophotometer (Multiscan FC, Thermo Scientific, Vantaa, Finland) for 40 min. We measured the absorbance at 492 nm every 25 s. The enzyme activity was determined according to the slope of the linear phase of the reaction (200–675 s after the reaction's start). In each run of the spectrophotometer we used 3 negative controls. We removed cases in which the absorbance curve was too irregular. The amount of PPO may correlate with body size. In order to correct for that, each slope was divided by the head width of each ant, photographed prior to the PPO measurement using a digital camera and a binocular.

### Statistics

#### Consistency in behaviour

Prior to analysis, we used the Z-score transformation controlling for the variance of the variables in different units, i.e., subtracting the mean and dividing by the standard deviation [36], and the transformed values were used in all further statistical tests. In order to test for consistency in behavior of the first and second trial, we used three different tests: (1) The Individual Stability Statistic (hereafter, ISS [37]): 
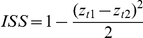
, where *z_t1_* and *z_t2_* are the values of the two trials of each behavior; (2) The Cronbach's α, measuring reliability of repeated tests [11]; (3) Pearson correlation. Higher values in all three tests point to high consistency in behavior while lower values indicate higher variance. We then used the Pearson correlation to correlate among all four ISS values (a single value for each behavior tested, based on the two trials). The purpose was to detect whether colonies showing consistent behavior in one trait are also consistent in other traits (i.e., ‘consistent in consistency’). It is plausible that colony size affects different behaviors and/or the level of consistency. In order to test for that, we correlated colony size with the four behaviors and the ISS levels.

#### Characterization of behavioral syndromes

We used the raw data (not transformed) to calculate the mean of the two trials documented for every behavioral trait (note that means enabled intermediate levels of relocation behavior, seven levels in total). Then we used the Z-score transformation on those mean values and performed a Factor Analysis (FA) on the four behavioral traits [36]. We also applied a varimax rotation to facilitate interpretation. Time to dead conspecific removal was given in negative values, so fast removal will score highly, similar to high aggression and high levels of nest reconstruction (high values represent stronger expression of each behavior). FA is a common way to characterize behavioral syndromes [11], [38]. The factors with eigenvalues larger than one should represent sets of behaviors. To further investigate these sets, we used a Pearson correlation.

#### Linking behavior with productivity and immune defense

We aimed at testing how the behavioral traits are linked to an important fitness component, the per-capita productivity. We already know that there is a negative link between colony size and per-capita productivity [28]. We therefore did not correlate the 1^st^ and 2^nd^ factors with per-capita productivity directly, but with the residuals of the linear regression of colony size and per-capita productivity (hereafter ‘productivity residuals’), in order to control for colony size. Then we used a two-way regression with the 1^st^ and 2^nd^ factors and their interaction as explanatory variables and the productivity residuals as the dependent variable. Regarding the immune defense level, we first tested whether colonies and ants performing different tasks differ in the PPO levels (a two-way ANOVA). Then, we used the average value of PPO for each colony (over six workers) and correlated the PPO levels with all four behavioral traits, using Bonferroni correction for multiple comparisons. We used STATISTICA v. 9.1 (StatSoft.Inc, Tulsa, OK, USA) and SYSTAT v. 11 (SYSTAT Software, San Jose, CA, USA) for all statistical analyses. See (Table S1) for the raw data of all experiments.

## Results

**Figure 3:**
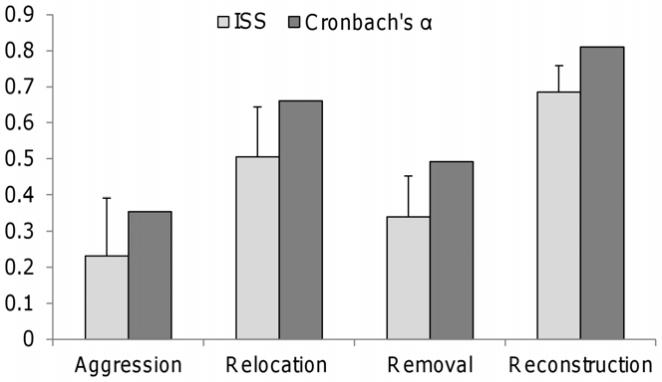
Consistency values (ISS; bright grey, left) of the two behavioral trials for each of the four behaviors measured (means±1 SE), and Cronbach's α coefficients of reliability tests (dark grey, right). High values represent a higher consistency level.

Consistency in behavior between the two trials, represented by the Individual Stability Statistic (ISS), was lower for aggressive behavior and corpse removal compared to nest reconstruction and relocation (Fig. 3). There was no correlation among consistencies (P>0.26 for all pairwise comparisons), meaning that there was no clear relationship among the consistency levels shown by colonies for different behavioral traits, i.e., high consistency in one behavior was not associated with high consistency in another one. When correlating pairs of trials separately for each behavior, significant positive correlations were evident for all behaviors except for aggression (aggression: Bartlett χ^2^ statistic = 2.27, df = 1, P = 0.13; nest relocation: χ^2^ = 13.36, df = 1, P = 0.0003; corpse removal: χ^2^ = 5.38, df = 1, P = 0.020; nest reconstruction: χ^2^ = 29.14, df = 1, P<0.0001). Cronbach's α tests produced similar results (Fig. 3), in accordance with the prior analysis using ISS values. Colony size was not correlated with any of the observed behaviors (P>0.32) nor with behavioral consistency (ISS values; P>0.39), even without a Bonferroni correction, suggesting no link of colony size with the behaviors observed. Table 1 presents summary statistics for the two trials of the four behaviors observed.

**Table 1:** Summary statistics for the two trials of the four behaviors observed.

Test	Mean ± 1 S.D.	Median
Aggression I	0.2401±0.2821	0.1235
Aggression II	0.3282±0.2528	0.2248
Nest relocation I	0.70±0.95	0
Nest relocation II	0.76±1.10	0
Corpse removal I	12.24±8.29	10.51
Corpse removal II	10.22±8.42	6.77
Nest reconstruction I	0.5295±0.3107	0.5531
Nest reconstruction II	0.3197±0.3179	0.2412

‘I’ and ‘II’ stand for the first and second trial.

**Figure 4:**
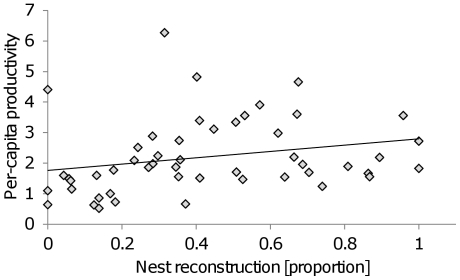
The positive relationship between nest reconstruction (proportion) and per-capita productivity (total number of brood divided by number of workers). The trend line is the best-fitted line according to a linear regression test.

We performed factor analysis (FA) on transformed means of the four behavioral traits. Eigenvalues, the percentage of the variance explained and factor loadings for different behaviors on all the FA axes are presented in Table 2. The 1^st^ factor represented an ‘aggressive vs. emigration-prone’ colony personality, because aggression and nest relocation had high loadings on this factor, but in opposite signs (positive and negative respectively). Corpse removal loaded positively, similar to aggression but to a lesser extent. We performed a Pearson correlation between the untransformed means of the two main behaviors of the 1^st^ factor, aggression and nest relocation, that confirmed to some extent the negative relationship (a marginally non-significant trend: Bartlett χ^2^ statistic = 3.12, df = 1, P = 0.077). The 2^nd^ factor was composed mainly of nest reconstruction (negative), while other behaviors showed loadings close to zero. The two other factors, the 3^rd^ and 4^th^ ones, had eigenvalues lower than 1 and are not further discussed. The 2^nd^ factor negatively correlated with the productivity residuals (coefficient = −0.355, P = 0.019). It suggests a positive link between nest reconstruction and per-capita productivity (note that nest reconstruction scored negatively on the 2^nd^ factor, and therefore a negative correlation of productivity with the 2^nd^ factor implied on a positive correlation with nest reconstruction; Fig. 4). The 1^st^ factor showed a marginally non-significant negative correlation with the productivity residuals (coefficient = −0.264, P = 0.077). It might suggest a negative effect of high aggression and a positive effect of relocation tendency on per-capita productivity, but since it is not significant, this possible association is not further discussed. The interaction term was not significant (P = 0.94) and was removed from analysis; statistics for the whole model are: F_2,47_ = 4.59, R^2^ = 0.164, P = 0.015. In summary, the 1^st^ factor showed a trade-off between aggression and corpse removal vs. nest relocation, while the 2^nd^ factor was mainly composed of nest reconstruction. The correlation of the 2^nd^ factor with productivity suggests that reconstruction contributes to this fitness component, but a manipulation is required to better support this association.

**Table 2:** Results of the two first factors of the factor analysis performed on mean behaviors of two trials after applying a Z-score transformation and a varimax rotation.

	Factor 1	Factor 2
Eigenvalue	1.51	0.97
% var. explained	36.1%	25.9%
Aggression	**+0.711**	+0.055
Nest relocation	**−0.749**	+0.083
Corpse removal	+0.614	+0.294
Nest reconstruction	−0.022	**−0.969**

Eigenvalues are taken from the unrotated analysis. Factor loadings higher than 0.7 are shown in bold. Factors 3 and 4 had lower Eigenvalues (0.78 and 0.74) and were not included.

**Figure 5:**
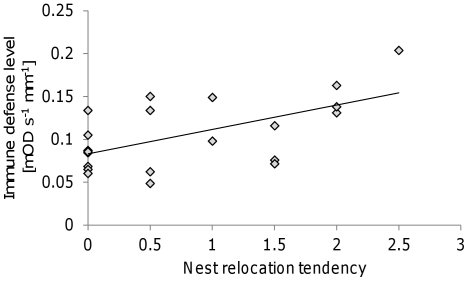
The positive relationship between the nest relocation tendency and the immune defense level, corrected for body size (head width of individual ants). The trend line is the best-fitted line according to a linear regression test.

Immune defense levels, represented by phenol- and pro-phenoloxidase (PPO), were evenly distributed among ants performing different tasks (F_2,56_ = 0.51, P = 0.60), but differed between colonies (F_20,56_ = 2.08, P = 0.017). The two-way interaction term was not significant (F_40,56_ = 1.08, P = 0.39). PPO levels were positively correlated with the relocation tendency (Pearson coefficient = 0.59, P = 0.020; Fig. 5), that is colonies which tended to relocate their nests show higher immune defense levels. All other behavioral traits were not correlated with PPO levels after Bonferroni correction for multiple comparisons.

## Discussion

Behavioral syndromes and personalities/temperament have often been demonstrated for solitary animals, but evidence for syndromes in insect societies or characterization of collective personality are still rare (but see [9], [10], [11]). Our study is one of the first to show collective personality on the colony level. The most important result is the evidence for a collective personality: colonies that defend their nest, either by fighting against intruders more aggressively or by removing infected corpses more efficiently, are less likely to relocate after a disturbance. It fits a common trade-off between competitiveness and emigration tendencies (e.g., [39], [40]). The behavioral consistency was the highest for nest reconstruction and relocation, less strong for removal of corpses and non-significant for aggression. This difference is probably related to the level of specialization each activity requires. Interestingly, the immune defense level was correlated with the nest relocation tendency, but with no other behavior, possibly because nest emigration is the only action exposing the whole colony to the surrounding environment. Finally, there is a positive correlation between per-capita productivity and nest reconstruction, suggesting a link between behavior and a fitness component.

The trade-off between territory defense, either by defending against intruders (elevated aggression) or parasites (efficient corpse removal), and the tendency to relocate is often shown by solitary animals. We suggest several explanations for this trade-off in ant societies. First, some nests might be considered to be of better quality than others and were therefore fiercer defended and less easily abandoned. *Temnothorax* colonies easily distinguish between nest types and often move if a better nest is available [14], [17]. Second, defending the nest implies that ants invested effort and energy, and therefore are reluctant to move out. Third, after colonies fail for some reason to fight back against intruders or to remove potential source of infection, they tend to relocate more readily to another nest, which might be more defensible. This trade-off between defense against intruders and relocation tendency has parallels in solitary animals owning a territory. For example, Cichlid fish males tended less to abandon their territory in the presence of predators if they were defending it before the encounter with predators [41], and less aggressive Cichlid females were more likely to emigrate than more aggressive ones [42]. Yet, the aggressiveness-relocation trade-off shown here also fits a more general ecological pattern: more competitive animals usually stay while less competitive ones emigrate, e.g., flour beetles [39], [40]. In related *T. longispinosus* colonies, aggressive colonies were more often found in dense areas [43]. This suggests that aggressive colonies remain in these dense areas and do not relocate despite frequent disturbances by intruders while less aggressive ones may move away.

The consistency in behavior was higher for nest reconstruction and relocation than aggression and removal of corpses. The two latter behaviors are performed by specialist ants, while the two former are a true collective behavior of the whole colony. Therefore, the repeatability of behaviors based on only a few specialist ants is possibly weaker, because specialist ants could have died between trials or on the other hand can also improve their efficiency with trials. Similarly, corpse removal in other social insects is done by few specialists, to minimize the exposure to contagious elements. This behavior may vary according to response thresholds to corpse/waste removal [19], [22], [44]. Similarly, aggression and kin discrimination are presumably carried out by a small group of specialist workers [45], leading to the same pattern of low consistency in behavior. In comparison, nest relocation requires a more coordinated effort: In a related *Temnothorax* species, one third of the colony recruits nestmates, actively participating in nest relocation [46]. We believe that the link between worker specialization and the consistency in colony behavior is important for further understanding behavioral syndromes in social insects. An interesting future direction would be to increase environmental heterogeneity and look for behavioral consistencies, expecting that consistency would be negatively correlated with environmental heterogeneity in space or time [47].

The immune defense level was positively correlated with the tendency of nest relocation, but not with any other behavior. Further research is required to establish more firmly the relation between immune defense level and relocation tendency, also in interaction with other environmental factors. We suggest that nest relocation is the only tested behavior exposing all colony members to the external environment, while the three other behaviors are carried out by a small fraction of the colony workers. Specialized workers can prevent the exposure of the whole colony to external risks in such cases, but exposure is inevitable during emigration. We suggest that colonies try to behaviorally adjust to the risk, but when not possible, react physiologically.

The relationship between different behaviors and fitness components is often taken for granted but is a fundamental issue in behavioral ecology (i.e., behavior is assumed to optimize fitness). Specifically in the field of behavioral syndromes and animal personality, there is a need for a better link with fitness [5], [26]. Smith and Blumstein [27] reviewed fitness consequences of animal personality, and showed that exploration was positively correlated with animal survival, and aggression increased with reproductive success. In social insects, Wray et al. showed a link between foraging and defense behaviors with productivity and survival [11], and Modlmeier and Foitzik demonstrated a positive relationship between the within-colony variance in behavior and productivity in the field [43] and in the laboratory [48]. We showed here a possible link between the nest reconstruction behavior and productivity.

Social insect colonies often prefer small entrances to their nests, as small entrances are more easily defendable (e.g., [14]). Nest usurpation of *Temnothorax* colonies is a common phenomenon, often by other ant species of larger colonies (e.g., [49]). Presumably to avoid invasions of different natures, *Temnothorax* species often reduce the entrance further more by accumulating dirt particles [25]. Other social insect species close their entrance in various ways in order to protect the colony against invasions [24], [50]. But to the best of our knowledge, this is the first study showing a positive correlation of nest reconstruction with some fitness component. Such a link can result from the positive contribution of this behavior to fitness or alternatively may indicate that colonies having more brood relative to workers block their entrance more intensively, because they have more to lose from invasions to their nests than colonies with less brood. The other observed behaviors did not correlate with per-capita productivity. A possible reason may be that after a while in lab conditions, behaviors which enhance survival and productivity under field conditions become less relevant. In general, measuring fitness of insect societies is challenging, because of their complex life cycle and long life span. It is also plausible that colonies would employ different short- and long-term strategies, resulting in various effects on fitness components [29]. Finally, a more experimental approach would be to investigate the suggested trade-off of aggression with nest relocation. Manipulating the nest condition/quality or alternatively inducing different levels of aggression can be a promising approach.

## Supporting Information

Table S1 **Raw dataset used for the statistical analysis.** For further information, please contact the first or second author.
(XLSX)
